# A Review of the Use of Natural Fibers in Cement Composites: Concepts, Applications and Brazilian History

**DOI:** 10.3390/polym14102043

**Published:** 2022-05-17

**Authors:** Diego Lilargem Rocha, Luís Urbano Durlo Tambara Júnior, Markssuel Teixeira Marvila, Elaine Cristina Pereira, Djalma Souza, Afonso Rangel Garcez de Azevedo

**Affiliations:** 1 Advanced Materials Laboratory (LAMAV), UENF—State University of the Northern Rio de Janeiro, Av. Alberto Lamego, 2000, Campos dos Goytacazes, Rio de Janeiro 28013-602, Brazil; diego.lilargem@gmail.com (D.L.R.); luistambara@gmail.com (L.U.D.T.J.); markssuel@hotmail.com (M.T.M.); elainecp@pq.uenf.br (E.C.P.); djsouza@uenf.br (D.S.); 2 Civil Engineering Laboratory (LECIV), UENF—State University of the Northern Rio de Janeiro, Av. Alberto Lamego, 2000, Campos dos Goytacazes, Rio de Janeiro 28013-602, Brazil

**Keywords:** lignocellulosic fibers, cement matrix, composite, natural qualities

## Abstract

The use of natural lignocellulosic fibers has become popular all over the world, as they are abundant, low-cost materials that favor a series of technological properties when used in cementitious composites. Due to its climate and geographic characteristics, Brazil has an abundant variety of natural fibers that have great potential for use in civil construction. The objective of this work is to present the main concepts about lignocellulosic fibers in cementitious composites, highlighting the innovation and advances in this topic in relation to countries such as Brazil, which has a worldwide prominence in the production of natural fibers. For this, some common characteristics of lignocellulosic fibers will be observed, such as their source, their proportion of natural polymers (biological structure of the fiber), their density and other mechanical characteristics. This information is compared with the mechanical characteristics of synthetic fibers to analyze the performance of composites reinforced with both types of fibers. Despite being inferior in tensile and flexural strength, composites made from vegetable fibers have an advantage in relation to their low density. The interface between the fiber and the composite matrix is what will define the final characteristics of the composite material. Due to this, different fibers (reinforcement materials) were analyzed in the literature in order to observe their characteristics in cementitious composites. Finally, the different surface treatments through which the fibers undergo will determine the fiber–matrix interface and the final characteristics of the cementitious composite.

## 1. Introduction

Fiber-reinforced composites have gained considerable acceptance in structural applications. The technological characteristics and low cost make them attractive to the cement industry. Natural fiber-reinforced composites are known to be an advantageous alternative and less harmful to the environment since they come from plants [1].

These composites have advantages over synthetic fibers, such as glass fiber composites. Glass fibers require greater energy consumption for manufacturing. On the other hand, natural fibers have biodegradability, non-toxicity, easy availability, non-abrasiveness, low density, low cost, good specific strength, and great resistance to corrosion and fatigue. However, natural fibers also have disadvantages such as loss of workability, fiber degradation, and material heterogeneity [2,3].

**Figure 1:**
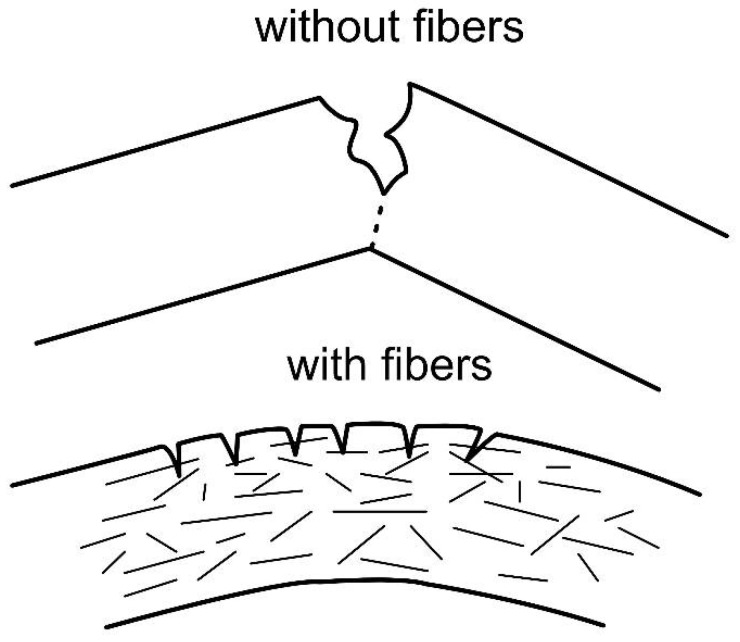
Difference between vegetable-fiber-reinforced concrete and one without incorporated fibers.

The cement composite reinforced with natural fibers can achieve mechanical characteristics superior to those of conventional materials already used in the industry. Fibers inhibit the initiation and propagation of cracks. They attenuate the progression of micro-cracks, thus preventing sudden rupture. As a result, the length of cracks in the hardened matrix is shorter, which considerably improves the impermeability and durability of composites exposed to the environment [4]. Figure 1 presents an image comparing concrete reinforced with vegetable fibers to one without incorporated fibers.

In the literature, several articles demonstrate mechanical and physical improvements with the incorporation of fibers in cementitious materials [5,6,7,8,9]. Geopolymer composites of fly ash with pineapple fibers showed an improvement in compressive strength, reaching 41.5 MPa and a flexural strength of 9.2 MPa. The reference without fibers presented a compressive strength of 12 MPa and flexural strength of 6 MPa [10]; coconut fibers treated in alkaline medium increase its ductility associated with the reduction in lignin with this treatment [11]; although it was difficult to homogenize with cementitious composites, the addition of 10–15 mm long jute fibers contributes to an increase in compressive, flexural, and tensile strength when it was equally distributed [12]; palm nuts showed an improvement in flexural strength [3]; palm fibers reduced the appearance of cracks due to the modification of the surface fiber after treatment [13]; açai fibers reduced the density of the hardened mortar and increased the flexural strength provided by a great interface between fiber and cementitious composite [14].

The final physical and mechanical properties of the hardened cement composite will depend on the proportion of fiber used, the type of fiber chosen, and the treatment used. These differences will directly affect the composite in the fiber and matrix interaction. Factors such as the roughness, length, and diameter of the fiber are fundamental for the efficiency of the bond with the matrix but not enough to guarantee the highest values of strength and stiffness for the composite [15]. The adhesion between the matrix and the reinforcement component exerts a strong influence on the characteristics of the composite because it is mainly responsible for transferring the efforts from the matrix to the reinforcement. A weak interface has an incomplete load transfer, which can result in the reinforcements being pulled out, resulting in composites with low mechanical strength [16].

To develop composites with good properties, it is necessary to improve the fiber–matrix interface and reduce moisture absorption. To ensure the durability of composites reinforced with vegetable fibers, these fibers must undergo surface modifications in order to infer better characteristics as a reinforcing material [17,18].

The chemical composition of the fiber also affects its performance. Fibers that contain large amounts of cellulose, hemicelluloses, and lignin have hydroxyl groups in their structure and tend to behave as a hydrophilic material. The combination of hydrophilic and hydrophobic materials in the processing of composite causes poor matrix–fiber interfacial adhesion, leading to ineffective stress transfer between the matrix and fiber and loss of final properties of composite materials. Chemical and physical treatments can improve the matrix–fiber interfacial bond, changing the surface polarity and increasing the fiber roughness, allowing better wettability of the fibers in the matrix [19].

Therefore, this work aims to review and situate the level of performance of the main natural fibers to consider using in cementitious composites. The characterization of the natural fibers, main surface treatment, compatibility with the cementitious matrix and future perspectives are presented to develop a cementitious composite.

## 2. What Are Natural Fibers?

Natural fibers are fibrous polymeric composite materials obtained from renewable natural sources, such as plants and animals. Vegetable fibers are composed of cellulose, while animal fibers are composed of proteins. These chemical and structural differences mean that cellulose and protein fibers react differently when exposed to heat and water [20]. Considering that extreme temperature changes can cause some protein structures to change permanently, these fibers end up being neglected in comparison with fibers of plant origin, which can go through the same processes without suffering damage [21].

Vegetable fibers have several advantages, such as providing low density and specific strength, and they are biodegradable and readily available from natural sources. In some cases, the fibers come from sub-processes of other industries, and in this way, the use of these fibers for new products takes advantage of a material that is rejected in the agro-industry, such as sugar cane bagasse, which is an important source of income for agricultural societies. Therefore, they have a low cost and a positive social impact. These plants are found in great abundance in Brazil, facilitating the search for raw material [22].

**Figure 2:**
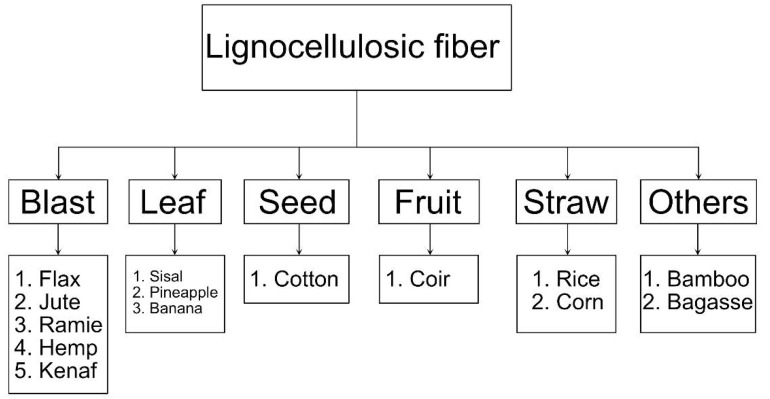
Classification of natural fibers by source.

Among the most used plant, kingdom fibers include flax, hemp, bamboo, sisal, and jute. Vegetable fibers can be classified morphologically according to the part of the plant from which they are obtained, as shown in Figure 2.

The fibers coming from the phloem, characterized as soft fibers, are generally: jute, flax, hemp and ramie [23,24]. Leaf fibers, characterized as hard fibers, are part of the fibrovascular system of the leaves [24,25]. Among leaf fibers, sisal is one of the most important and widely used and is abundant in many tropical countries. Among seed and fruit fibers, coconut or coconut fiber is considered the most suitable for concrete due to its original fiber length and higher durability in alkaline medium [26,27].

Kraft fibers, the main constituent of cardboard materials, are also considered natural fibers that contain cellulose fibers. They have been investigated in cementitious materials and can enhance its tensile properties [28]. Softwood and hardwood fibers are also incorporated in cementitious materials; however, softwood fibers can increase the flexural strength and toughness of the composite [29].

### 2.1. Lignocellulosic Fibers

Lignocellulosic fiber is a scientific name that refers to natural fiber because all plant fibers are made up of the constituents: cellulose, hemicelluloses and lignin. Most plant fibers contain 50–70% cellulose. Table 1 presents the main fibers studied in the literature and some fibers present in Brazil. Among the fibers with abundance in Brazil, pineapple, tucum and curaua fibers stand out with high cellulose content.

Cotton and pineapple are natural fibers with higher cellulose content, while coconut and piassava presented the highest lignin content, and kenaf has a structure containing more hemicellulose. Cellulose molecules form a rigid network, resulting in a compact structure. In plant cell walls, cellulose is organized in long and organized microfibrils, which results in large fibers. Hemicellulose molecules structure the cellulose fibers, and the lignin fills the spaces between the polysaccharides.

### 2.2. Biological Structure of Fiber

**Figure 3:**
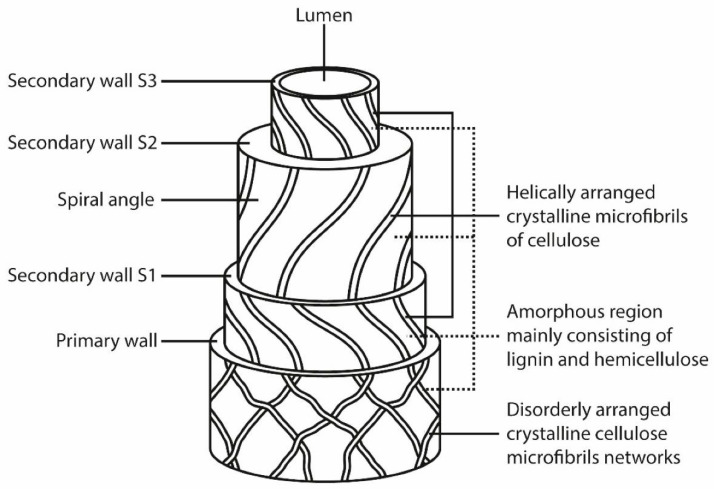
Cell wall structure [35].

The arrangement of macromolecular components in the plant cell wall is complex. It is organized by layers, and the fiber cell wall is not a homogeneous membrane. Figure 3 provides an example of the cell wall structure [34].

Cell fibers have a diameter of 10 to 25 µm and are made up of an outer layer called the primary layer. This layer is composed of a network of randomly arranged cellulose microfibrils (semicrystalline) connected to an amorphous phase of hemicellulose and lignin, which act as a matrix for the cellulose bundles [36]. Cellulose microfibrils with a diameter of about 10 to 50 nm provide mechanical strength to the natural plant fibers [37]. In the inner secondary, three layers are generally present, and in each one, the cellulose wall microfibrils are arranged helically in relation to the long axis of the fiber. In most natural plant fibers, the cellulose microfibrils are oriented at an angle to the normal fiber axis called the Microfibrillar Angle (MFA). The angle between each layer is different, with the microfibrillar angle in relation to the axis being an important factor that determines the mechanical properties of the fiber [38].

The inner secondary layer is composed of three secondary layers, secondary 1 (L1), secondary 2 (L2), and secondary 3 (L3). At L2, the microfibrils are aligned according to an angle θ, which is the angle between the fiber axis and the microfibrils [39].

Most of the cellulose is present in the L2 layer, precisely because it is thicker and has a higher microfibril content. The wall L2 corresponds to about 65 to 80% of the solid volume of the fiber. L1 and L3 are much richer in xylenes, especially the L3 layer, which is the richest in hemicellulose concentration (50 to 70% hemicellulose concentration) [40].

There is the middle lamella (M), which determines the mechanical properties of the fiber. This M layer consists of several “coiled cellular microfibrils” formed from long-chain cellulose molecules. The outer secondary cell wall (L3) has the same thickness as the primary cell wall and is composed of lamellae spiraling in opposite directions. The cell wall (L2) consists of the main volume of the cell, and this layer has important mechanical properties, such as the modulus of elasticity [41].

The properties of the fibers are determined according to the MFA, structure, cell dimensions, defects, and chemical composition (cellulose, hemicellulose, and lignin content) [42,43]. It is for this reason that different lignocellulosic fibers have such different physical characteristics [44].

Basic parameters such as cellulose content and spiral angle characterize the mechanical behavior of plant fibers. Higher cellulose contents in the fiber result in an increase in tensile strength. On the other hand, if the spiral angle of the fibers decreases, the tensile strength increases [45]. The amount of cellulose is closely related to the crystallinity index of the fiber and the MFA with respect to the main fiber axis. It has been shown that fibers with a high crystallinity index and/or cellulose content have better mechanical properties, i.e., the MFA determines the stiffness of the fibers, which in turn determines the mechanical properties of the composite. A low MFA makes the fiber stiffer, less flexible, and mechanically stronger [46].

## 3. Properties and Importance of Use in Cementitious Materials

Recent studies report the mechanical properties, structural behavior, and possible applications of natural fibers in cementitious materials [14,31,32,47]. Several factors affect the properties of natural fiber-reinforced composites, such as the proportion of cellulose, as presented previously, the type of fiber used, fiber geometry, fiber structure, fiber surface (related to its adhesion and interface with the hue), mixing method, fiber dispersion (avoid fiber agglomeration, reduces the presence of voids and favors the interfacial bond with the matrix), fiber orientation (the alignment to a parallel direction contributes to a better viscosity of the matrix), matrix selection, interface strength (matrices of cementitious materials and natural fibers, which are limited due to the hydrophobic characteristic of natural fibers; however, treatments can improve the interface strength), manufacturing (temperature, pressure, and speed of processing of the fiber or composite), porosity, curing method, physical properties, cell dimensions, and microfibrillar angle [48,49,50,51].

The strength and stiffness of the fibers depend on the cellulose content and the spiral angle that the microfibrils of the inner secondary cell wall have with the fiber axis [52]. High cellulose content and low microfibril angle are desirable properties of a fiber to be used as reinforcement in cementitious composites. Fiber diameter and length affect strength. Fibers with larger diameters tend to confer more compressive and flexural strength to composites, as observed in composites with sisal and bamboo fibers [53,54,55].

As each fiber has different characteristics, as shown in Table 1, these same fibers have different physical and mechanical properties, as can be seen in Table 2 [24,56]. It is also important to mention that as these fibers come from plants that grew in different circumstances, there are high variations in their properties.

Comparing Table 1 and Table 2, it is noted that some fibers with a high percentage of cellulose present high values in mechanical properties, tensile strength, and Young’s modulus, such as kenaf and hemp, respectively. Açai fibers presented significant values of cellulose; however, the heterogeneity of the natural material presents a reduction in the values of tensile strength.

When comparing natural fibers with some synthetic fibers, as seen in Table 3, it is observed that lignocellulosic fibers have specific mechanical properties with similar values to those of synthetic fibers, and in some cases, higher. The mechanical characteristics of natural and synthetic fibers are similar, and due to this, the application of natural fibers can benefit the environment and the properties of composite materials [58].

Synthetic fibers have higher tensile strength and elasticity than natural fibers. However, when analyzing the specific modulus and specific tensile strength, natural fibers can outperform synthetic fibers, such as metallic and glass fibers.

Commonly used in civil construction, steel fiber [59] presented a higher tensile strength and elastic modulus when compared with the natural fibers (Table 3). However, the high density, cost, and ease of agglomeration during the mixing of the metallic fiber can negatively affect the properties of mortars and concretes. Therefore, for applications that require a material with higher strength and lower weight, the use of concrete reinforced with lignocellulosic fiber is of greater interest.

Fiberglass presents a lower density than metallic fiber and higher tensile strength and elasticity than lignocellulosic fibers [60]. The maximum specific tensile strength of curaua and pineapple fibers manages to overcome, at some points, fiberglass, which has its minimum value of 0.69 MPa.

In addition to the type of fiber used, some general characteristics of all fibers affect the properties of the composite. Fiber geometry is intrinsically linked to composite quality. The longer the fiber length or the smaller its diameter, the more the fiber aspect ratio length/diameter (l/d) improves, which has a positive effect on the mechanical properties of composites [61]. Nonetheless, decreasing the fiber length more than the critical length (smallest possible length for load transfer) implies a reduction in the transfer of tension from the matrix to the fiber since short fibers are not the most indicated for reinforcement material [62].

Regarding the orientation of the fibers concerning the applied load, the best mechanical properties of composites are obtained when the fiber is aligned parallel to the load. Large reductions in tensile strength and modulus of elasticity are observed with an increase in the fiber orientation angle to the main loading direction (on-axis direction) [63].

The increase in the percentage of fiber bulk properties leads to the higher mechanical strength of the composite material; nevertheless, there is a limit to this. From a certain value, which will vary for each material, the addition of fibers will reduce the characteristics of the composite [64]. Water absorption is increased with a greater amount of fiber, leading to swelling of the natural fibers and detachment of the fiber–matrix interface, as well as deteriorating the mechanical properties of the composite [27]. This implies a reduction in the durability of the cementitious composite due to the decrease in mechanical strength and tenacity. This causes a reduction in the pull-out strength of the fibers due to the combination of the weakening of the fibers by the alkaline attack, caused due to the cement hydration, the fiber mineralization, and the volume variation due to its high water absorption [65].

Extensive studies were conducted to find the optimal relationship between volume fraction and ideal mechanical properties. In cement matrices, the proportion of fibers hardly exceeds 5% [53,66,67]. Studies with higher percentages show little wettability of the fibers concerning the matrix, resulting in a drop in the mechanical characteristics [13].

Ensuring a good dispersion of the fibers in the matrix provides better results, reducing the void content, and also leads to an increase in the interfacial bond between the matrix and the fibers [68].

### 3.1. Compatibility between Fibers and Matrices

What will define the success of a composite with fiber is the interface between the fiber and the matrix, i.e., the quality of the bond between the matrix and the fibers. From this, the quality of the load transference from the brittle cement matrix to the ductile reinforcement is obtained [20].

The weak fiber–matrix interaction is one of the main problems related to the manufacture and use of composites reinforced with natural fibers. Lignocellulosic fibers are hydrophilic and may increase fiber deterioration mechanisms. Deterioration causes low interfacial surface tension, and as a result, load transfer to the reinforcement is incomplete. The agglomeration of fibers during processing resulted from the non-homogeneity in the cement matrix, which interferes with the equality of characteristics throughout the composite. Finally, cement itself is an alkaline medium that facilitates fiber degradation and composite durability [69].

Even though natural fibers present these negative points, there are currently many different strategies to improve fiber–matrix adhesion. The chemical or physical modification of the fiber surface, through surface treatments, tends to improve the compatibility between the fiber and the cement matrix [70]. The fiber treatment is discussed in Section 4.

Ruslan et al. [71] evaluated the effect of synthetic fiber as reinforcement of cementitious materials. The authors suggest the theory of Schklowsky-De Zhen to predict the optimum fiber content, evaluating the rheological parameters and physicomechanical properties of concretes. In this work, polypropylene fiber is recommended with a volume of 0.45%. A higher concentration of polypropylene fiber results in an agglomeration, and the strength of the reinforced material is determined by the rupture of the fiber. Natural fibers can be added to up to 6% of the paste volume. However, it is known that increasing the percentage of natural fiber results in an increase in the matrix fiber transition zone, which causes an increase in porosity and a reduction in the mechanical strength of the mixture [72].

Table 4 presents an overview of the properties and conditions of different fiber/cement systems. Some authors do not recommend curing in water due to the hydrophilic behavior [5]. NaOH treatment showed an increase in the crystallinity of the fiber and a better interface with the matrix [1,33]. Treatment of 1% Na_2_CO_3_ for 7 days resulted in an increase in the split tensile strength of the sisal fiber, while 1% fiber in concrete composites presented an increase in mechanical properties (compressive and flexural strength) [53].

### 3.2. Comparison of the Characteristics of Conventional Cement with Lignocellulosic Fiber Composites

The fibers used in the concrete act as barriers and prevent the development of cracks. These fibers are distributed and arranged uniformly, thus increasing the energy absorption capacity and ductility of the concrete. In addition, it can help the structural integrity and cohesion of the cementitious material [4,73,74].

Synthetic fibers were widely used for this purpose. However, to improve the concrete characteristics and make its manufacture more sustainable, natural fibers began to be studied. In this section, the contextualization, mechanical, and durability properties provided by the main Brazilian fibers used in concrete will be presented.

#### 3.2.1. Sugarcane Bagasse

Sugarcane is one of the most characteristic plants in Brazil. It is used for the production of alcohol and sugar, but it generates a lot of bagasse as leftovers from the process [75]. This bagasse has already been used to generate energy in other research [76]. In recent research, sugarcane bagasse was used as complementary cementitious material [77] and as fiber in cementitious composites.

Bayapureddy et al. [77] performed compressive strength, split tensile, and water absorption tests on a composite with sugarcane bagasse ash in cement in the proportions of 5%, 10%, 15%, and 20%. An increase in tensile and compressive strength was observed at the levels of 5, 10, and 15% of sugarcane bagasse ash in the cement, but the strength was reduced by 20%. Demonstrating that 15% is the maximum point for ash increment, the same result was found by Berenguer et al. [78], who analyzed the durability of concrete structures with sugarcane bagasse ash. The addition improved the mechanical properties of the concrete. As for water absorption, it was observed that it was reduced in all samples containing sugarcane bagasse ash compared to conventional cement [77].

The treatment of the sugarcane bagasse fibers was evaluated to produce Portland cement composites [79]. A hot water pre-treatment (100 °C for 30 min) was compared with natural fiber. The authors observed that the non-treated fibers delayed the Portland cement hydration due to the presence of extractives and impurities. The pre-treatment reduced the effect of the delay of hydration and consequently increased the mechanical properties of the composites. The hot treatment also increased the contact angle of the fiber, reaching values higher than 90°. This result showed greater difficulty in forming a monolayer of water on the fiber surface and less water adsorption.

#### 3.2.2. Coconut

Coconut fiber is a common fiber in Brazil. A large amount of research is related to incorporation into mortar [11,80,81,82,83]. Danso and Manu [81] analyzed the differences in the characteristics of soil–cement mortar composites by increasing the content of incorporated coconut fibers and the amount of lime in the composite. The authors observed that the concrete with the addition of 0.2% coconut fiber and 5% lime content in the mortar showed higher density, compressive strength, and tensile strength. The same behavior was observed with other works with coconut fibers [11,84].

To attenuate the propagation of cracks in high-performance marine concrete structures, it is possible to incorporate coconut fibers into the composition of composite materials. Experimental results showed that the addition of coconut fibers improved the compressive and flexural strength of the structures by up to 13% and 9%, respectively. However, in terms of durability, chloride penetration, intrinsic permeability, and carbonation depth increased with increasing fiber content. The authors recommend that coconut fibers be treated before their use in concrete to protect them from degradation [85].

To increase the durability of composites with coconut fiber, some studies [84,85] indicate the use of glue or latex to decrease the ion transportation in the aqueous medium that fills the pores of the cement paste, adsorbing portlandite in the fiber that can provide local pozzolanic reactions in the interior of the cementitious matrix, which can protect the coconut fiber surface from the alkaline attack.

A study demonstrated that although the addition of coconut to gypsum mortar does not improve the strength of the masonry, it presents a significant improvement in the post-cracking behavior, such as residual strength, residual strain energy, ductility, and flexural toughness [82]. Coconut fiber treatments also can increase durability in composites. Silva et al. [83] evaluated four types of treatment using silica fume and metakaolin as a coating agent for the coconut fiber to obtain a coconut fiber-cement composite. The authors observed improvement in the flexural strength and durability of cement-based composites.

#### 3.2.3. Sisal

The sisal fibers are obtained from the extraction of sisal leaves usually performed by semi-automatic raspadors. Its microstructure is formed by numerous individual fibers (fiber-cells) that are about 6–30 μm in diameter [86].

Cementitious composites reinforced with sisal fibers treated with Na_2_CO_3_ in an M30 concrete mix showed an increase in compressive strength of 3.5%, for a proportion of 1% of fiber, when compared to conventional concrete. Seven days of treatment with Na_2_CO_3_ results in a large number of Na^+^ and CO_3_^2−^ ion deposited onto the fiber surface that chemically reacts with the Ca^2+^ present in the cement, resulting in a gain of compressive and tensile strength [53]. The concentration of 1% of sisal fiber was considered by Mithun et al. [53] as the ideal dosage for this type of fiber. However, Iniya et al. [87] observed a reduction in compressive strength when the sisal fiber treated with NaOH was increased. However, if the fiber reduced the compressive strength, it slightly increased the flexural strength in proportions of 0.5% and 1%.

Okeola et al. [88] analyzed the physical and mechanical properties of sisal fiber reinforced concrete and observed that the workability of the mortar in the fresh state decreases when sisal fiber is added to the mixture due to the hygroscopic properties of sisal. Porosity and water absorption were increased when fiber was incorporated and became greater for higher fiber contents.

#### 3.2.4. Curaua

The curaua fiber presents mechanical and physical properties with potential for use as construction material composites for the cladding and facades of buildings due to its lightness and low conductivity [57]. These composites also show an increase in toughness and matrix–fiber stress transfer when 2% curaua fiber is included in the mix [89].

In addition to low density, Teixeira et al. [89], noted that with the increase in fiber content, 2% fiber, the composite showed an increase in absorption due to the greater number of fibers per unit volume, as well as the filling of the matrix pores. Because of this, the use of short curauá fibers (25 and 50 mm long) as a reinforcement of cementitious mortar can produce composites with adequate mechanical properties that allow their use in semi-structural applications [90].

#### 3.2.5. Pineapple

Pineapple-fiber-reinforced geopolymer concrete demonstrated an increase in compressive strength for different percentages of fiber regarding the weight of the air-plaster [10,91]. For conventional concrete, Abirami et al. [92] noted an increase in compressive strength by up to 30.62% with the addition of 0.1% pineapple leaf fiber. The flexural strength was increased by up to 46% over conventional concrete, while the tensile strength was increased by up to 14.20% by adding the same fiber content.

Regarding water retention, de Azevedo et al. [67] observed that it increases with the fiber content added, both treated and untreated fiber. The incorporation of treated fibers up to 5% results in the maintenance of workability properties in mortars.

#### 3.2.6. Jute

Jute fiber composite has reduced workability and higher compressive strength for different types of concrete (M25, M30, and M40) [93]. The flexural strength is also increased as more fiber content is incorporated until 0.25% with the fiber cut length of 10 and 15 mm [12]. However, Zakaria et al. [12] observed an unexpected drop in compressive strength due to the inclusion of longer wires (20 and 25 mm). For greater length and greater proportions of reinforcing material, a lower load-bearing zone is created throughout the composite. Zárate et al. [94] reported that fibers that are longer and larger in volume curled up during the blending process.

#### 3.2.7. Piassava

Untreated piassava fibers were used for the reinforcement of lightweight concrete containing 4% and 6% of ethylene-vinyl acetate to improve the flexural strength of the material [95]. It was observed that the addition of piassava fiber results in reduced compressive strength, but the presence of fiber has a positive influence on tensile strength.

Regarding water absorption, da Fonseca et al. [31] noted that compared to other fibers, such as tucum palm and jute, piassava fiber has the lowest water absorption. Regarding compressive strength, the piassava composite with 4.5% fiber had a slight increase in strength from 22.23 to 24.59 MPa [31].

Reis and Motta [96] noticed that small amounts of piassava fibers, 2%, reinforce polymeric castor mortars in terms of compressive strength. As for crack propagation resistance, piassava fibers play an important role in significantly improving fracture toughness and stiffness for 1% piassava fiber content.

#### 3.2.8. Açai

**Figure 4:**
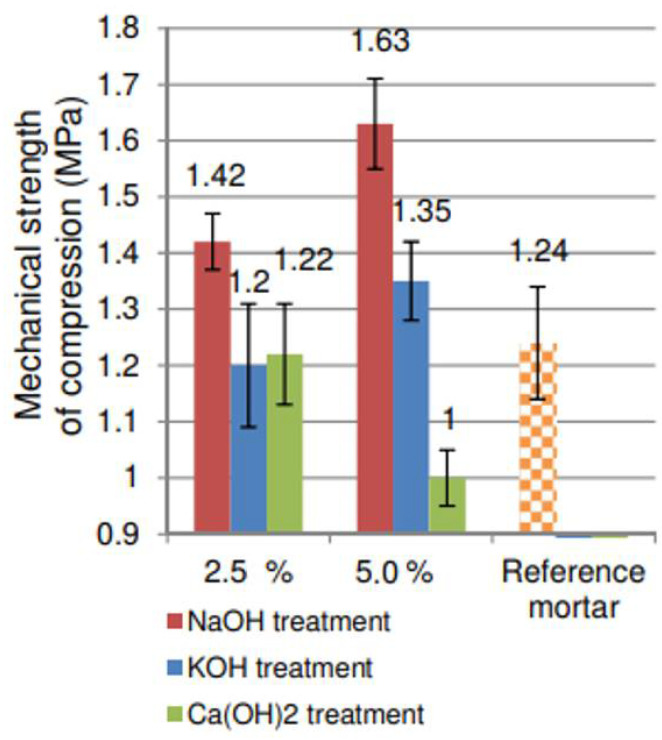
Results of the mechanical strength of compression of each methodology of treatment in reference [97].

Açai fibers are a potential option for strengthening cementitious materials, especially for countries, such as Brazil, that have high productivity of products that use this fruit and have several agro-industrial activities where several açai fibers are rejected. The studies on açai fibers are very recent. Azevedo et al. [33] analyzed the behavior of açai fibers mixed with cement, lime, sand, and water. The composites showed an increase in compressive and flexural strength, following the trends of other fiber-reinforced composites. The fibers were treated with a 5% NaOH concentration in the water mass. The flexural strength was increased from 1.19 MPa (with 0% fiber) to 1.76 MPa (with 3% fiber). The compressive strength increased from 3.52 to 4.23 MPa, also with 3% fiber content [33]. When fiber content increased by 3%, the value of strength was reduced. Azevedo et al. [33] concluded that 3% fiber achieved the ideal wettability for açai fibers in this composite. Rocha et al. [97] analyzed the compressive strength of the composite with açai fibers for proportions of 2.5% and 5%, treated with NaOH, KOH, and Ca(OH)_2_. The treatment with NaOH presented the best results, followed by the treatment with KOH and Ca(OH)_2_, with values of 1.63, 1.35, and 1 MPa, respectively, with a fiber content of 5%. Figure 4 presents the values from [97].

**Figure 5:**
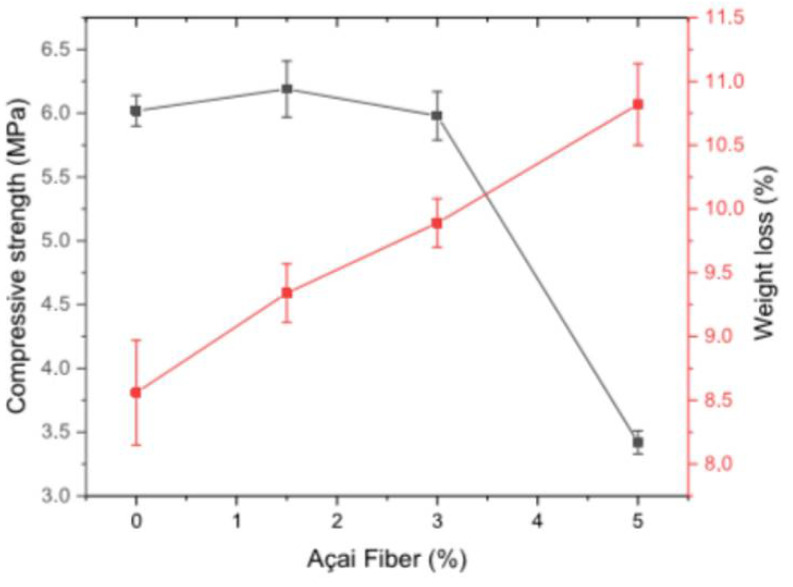
Results after salt spray attack [14].

Marvila et al. [14] analyzed the durability of açai fiber composite in a high alkalinity medium (such as Portland cement matrix) and obtained positive results up to 1.5% fiber content, resulting in higher durability properties when subject to drying cycles, salt spray attacks, and thermal shock tests. Figure 5 presents the results of the loss of mass and compressive strength of mortars after a salt spray attack [14].

## 4. Why Are Natural Fibers Treated for Application in Cementitious Matrices?

Lignocellulosic fibers undergo treatments because they have a very high hydrophilic character that tends to reduce the efficiency of the cementitious composite. The modification of the surface of the natural fiber is a necessary method to improve the fiber–matrix adhesion and, consequently, the properties of the composite. In addition, the treatments help to: remove impurities from ash, waxes, and sugars that can delay the hardening of the fibers, improve adhesion, increase crystallinity and consequently mechanical strength, or even improve fiber durability aspects [98,99,100,101].

**Figure 6:**
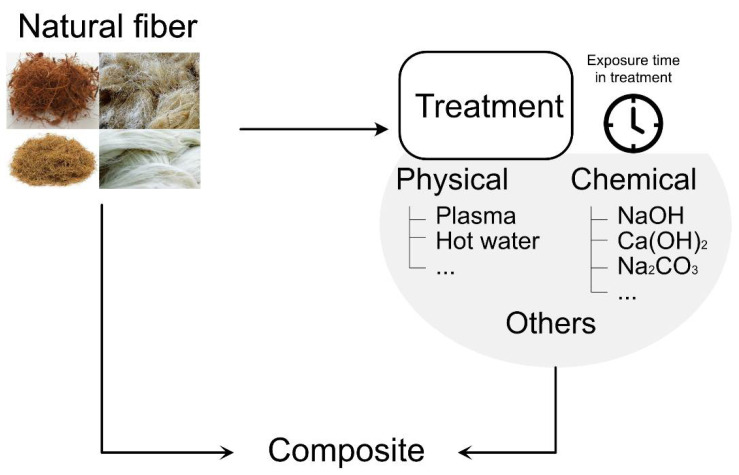
Natural fiber treatment scheme.

There are different ways of modifying the surface, and this chapter will review some essential points and demonstrate some new and alternative methods that aim for even better results. A summary of the treatment fiber for application in composites is shown in Figure 6, and the treatment procedure is described in the following topics.

### 4.1. Chemical Treatments

The most used fiber surface modification in the literature is chemical treatment [102]. Chemical treatment can be acetylation, silane treatment, and mercerization [103]. Several studies demonstrate the use of chemical surface treatments on cellulosic fibers to reduce their hydrophilic character and improve their adhesion properties with the matrix [104,105,106]. Most works with lignocellulosic fibers in cementitious matrices are based on treatments with alkaline solutions: Ca (OH)_2_, NaOH, KOH, LiOH, Na_2_CO_3_ [14,53,97,106,107,108,109]. The chemical treatment involves modifying the fiber’s hydroxyl groups and introducing other interacting groups that effectively interconnect with the matrix at the interface.

Santos et al. [105] compare the difference between mercerization treatments with NaOH and Ca(OH)_2_ to piassava fibers. Treatment with calcium hydroxide proved to be less aggressive to the surface of a natural fiber than treatment with NaOH. Sedan et al. [110] observed a positive effect of treatment with 6% NaOH (in relation to water weight) on the flexural strength and fiber–matrix adhesion of composites with a fiber content of 16% by volume. Kaplan and Bayraktar [111] observed that hemp fibers treated with NaOH resulted in an increase in compression from 24% to 43%, and flexural strength from 19% to 23%, before and after the wetting and drying cycles. Adhesion strengths were especially improved with NaOH treatment in several other studies [112]. Regarding the concentration of the hydroxide used, Beltrami et al. [102] analyzed concentrations of 1%, 5%, and 10% of NaOH in curaua fibers and observed that the alkaline solution in low concentration (1% NaOH) did not promote an effective improvement in the adhesion of the fiber to the matrix, not significantly altering the mechanical properties of composites. On the other hand, treatment with a high concentration alkaline solution (10% NaOH) weakened the fiber structure, which reduced the mechanical properties of the composites.

Chemical treatments by mercerization are performed by placing the fibers in a bath of chemical solutions that remove or add components to the surface. To reduce the hydrophilic character, it is necessary to reduce the number of reactive hydroxyl groups, resulting in reduced water absorption. Concomitantly, the formation of bonds between the cellulose fibers and the cement matrix is necessary to improve the mechanical properties of the composites. The use of silicon-derived coating agents showed promise in obtaining efficient adhesion between the fiber and the cementitious matrix and a significant reduction in the hydrophilic character of the fibers [106].

Alkaline treatment, by removing chemical constituents from a fiber, such as a hemicellulose, lignin, and pectin, increases the roughness topographies of the fiber surface. Physically, the presence of a rough surface provides a mechanical interlock between the fiber surface and the matrix, which can improve the interfacial bond between them [68,113]. In addition, the alkaline treatment modifies the cellulose structure, increasing its crystallinity and reducing impurities such as sugar, which acts as a setting retardant in mortars [67].

**Figure 7:**
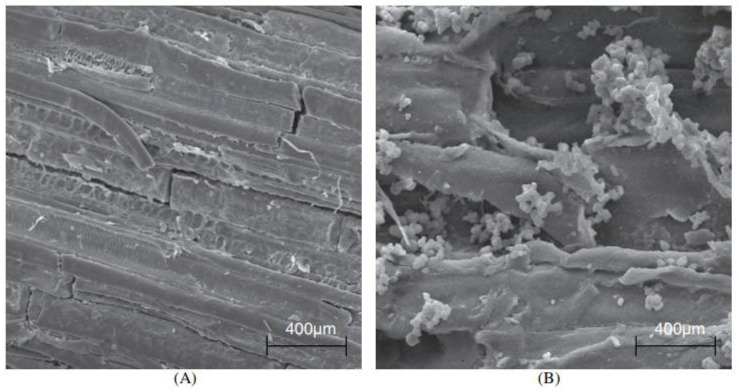
Morphology of sugarcane bagasse fibers: (**A**) untreated; (**B**) treated [114].

Figure 7 shows sugarcane bagasse fiber before and after treatment. Note that the elimination of the surface layer and the presence of materials dispersed in the fibers are caused by the modification. Due to this, there is an increase in the roughness of the fibers, which results in a better interfacial bond between the fibers and the matrix [114].

However, chemical modifications present two main problems: extensive modification of the fiber and reaching its interior reduces the mechanical properties of the fiber as a whole; and the generation of large volumes of waste, which cannot be easily discarded as they are harmful to human health and the environment (or requiring water washing for correct disposal). This increases the cost of these processes due to the steps necessary to neutralize or treat the waste generated [65].

Because of this, other types of treatments have been developed. The proposal is to find even more efficient ways to improve the fiber–matrix interaction than in chemical treatments.

### 4.2. Physical Treatments

A new type of treatment that has been used in natural fibers is physical treatment. Unlike chemical treatments, physical treatments do not affect the bulk properties of the fiber but rather cause physical changes on the surface of the lignocellulosic material due to the energy deposited in it. Another difference is that the physical treatment does not generate any type of fluid that is harmful to the environment, as in chemical treatments.

The plasma (ionized gas where electrons and free ions coexist) reaches the fiber, neutralizing some degradation mechanisms through the removal of electrons from atoms and molecules of the lignocellulosic fibers. Physical treatment can use cold plasma or corona plasma, which work in similar ways, their main difference is the way it is generated.

Barra et al. [98] treated sisal fibers in a cold plasma vacuum reactor using methane gas. The authors observed that the amount of water absorbed by the treated fibers is lower than that of the untreated ones. Plasma polymerization of CH_4_ deposited a very smooth hydrocarbon layer composed of CH_2_ and CH_3_ groups [115]. The use of plasma has been used to reduce the hydrophilic character of natural fibers [116], however, for a different matrix than cement [117]. This type of treatment for this type of matrix is very recent.

However, the tensile strength of the treated compounds was only improved by about 4% compared to that of the raw compounds. In addition, the Young’s modulus of the composites has been increased by 70%, but this method requires a lot of energy during preparation [118].

### 4.3. Other Treatments

Other treatments aim to coat the lignocellulosic fibers with materials of natural origin, similar to what is achieved in mercerization, but with products that are less aggressive to the fiber, such as coating beet pulp with sucrose [119], resulting in a better interfacial zone between the treated particles and the cement. Page et al. [120] coated natural fibers with linseed oil, which hardens by oxidation in contact with air, forming a solid film. It was observed that the treatment was successful at decreasing the water absorption when compared with the reference mortar (without fiber) and other mortars treated by plasma and mixed with blast furnace slag.

Simpler treatments also demonstrate success in improving the interfacial interaction between the fiber and the matrix, such as the hot water wash treatment performed by [99]. Fonseca et al. [99] compared the fiber washing treatment with three other treatments, such as hornification, alkaline treatment with NaOH, and hybridization. Hornification consists of the application of ten cycles of wetting/drying, as performed by [121]. The hybridization process consisted of a combination of the chemical and physical treatment of the fiber, such as the washing in hot water, followed by treatment with NaOH and an application of a 5% hydrogen peroxide solution for 3 h in a fiber solution ratio of 1:20 [99]. The hybridization process contributed to increasing the mechanical strength and crystallinity of the fiber.

Fonseca et al. [99] observed that chemical treatments (NaOH solution and hybridization) resulted in greater water absorption. The hornification treatment alters the surface of the cellulose fibrils, promoting less water absorption, a result also found in [122], while the hot water treatment results in less water absorption due to changes in the fiber structures, which have become more rigid.

Biological treatments use bacteria and fungi to change the surface of the fiber. The process is carried out in aqueous environments and has a relatively low cost of execution, but regularly, it consumes more time and pollutes the water in nature [123].

A polyelectrolyte solution also can be used to modify the surface of natural fibers [124]. This method enables the formation of an adsorptive layer on the surface of natural cellulosic fiber. In this method, the polyelectrolyte self-assembly layers deposit on the surface of the fiber due to the electrostatic action of hydrogen bonding. As a result, it is possible to improve the heat resistance of the fibers, mechanical properties, water resistance, and the bonding force with the matrix.

## 5. Effect of Fibers

The type of fiber influences the final characteristics of the composite; however, it is not the main characteristic of its success or failure. Even fibers with high cellulose content and smaller helix axis angle can result in composites with lower strengths. This is due to the heterogeneity of the fibers, and fibers from the same source may have different percentages and cellulose from each other.

The main point for a composite to work well is the interaction of the fiber and the matrix, and for natural fibers, a good interface will be achieved according to the type of surface treatment performed on the fiber. To compare some results, Table 5 shows some compressive strength results after 28 days of curing for several cementitious composites with different lignocellulosic fibers from Brazil, with different fiber insertion contents, and with different treatments or without any kind of treatment.

Table 5 shows the results of cement composites made from sugarcane, coconut, sisal, açai, pineapple, and piassava. Some of these fibers, such as açai, pineapple and piassava, underwent some treatment before being inserted into the matrix, while the others were inserted in nature. It is observed that even some treated fibers obtain results below those that have undergone some treatment. However, this should not be seen as a detriment of the surface treatment but rather that that type of fiber can interact better with the matrix than some treated fibers. This is what happens, for example, in coconut fiber. Even without treatment, its content of 0.2 was higher than the other fibers. Below that, the acai fiber content of 3% achieves a relative gain value of 120.2%, the highest among treated fibers.

It is also noteworthy that after some fibers reach their maximum RCI value, they decrease to values higher than the reference trace, which is what is noted for coconut fiber (0.8% to 115%), açai (4.5% to 111.9%) and pineapple (0.2% to 108.8%), demonstrating the fiber reinforcement effect in the cement matrix, even outside the ideal value.

The surface treatment can only be observed in the same fiber, and only in this way is it possible to compare if there was an improvement in the properties. The work of Azevedo et al. [33] observed efficiency in comparing treated and untreated fibers. In addition, all composites with some acai fiber content, as studied by [33], had higher compressive strength results than the reference concrete (shown in Table 5 as the percentage with 0% fiber). All six fibers presented in Table 5 obtained higher values of mechanical strength as the fiber was inserted when compared to the one with 0%. It was also observed with the behavior highlighted in previous chapters that from a certain percentage, the values of the compressive strength fall. They are different for each fiber. The sugarcane bagasse composite reaches its maximum value in the proportion of 15% [77], the coconut 0.2% [81], sisal with 1.2 [65], açai with 3% [33], the pineapple with 0.1 [92], and the piassava with 1.5 and 4.5 [31]. Note that, except for sugarcane bagasse, all fibers are inserted in small proportions, up to 4.5%. The reason for the higher proportions of bagasse is that it was replaced by cement in the mix. As for the other mixtures, there was only an increase in a small amount of fiber about the mass of the cement.

**Figure 8:**
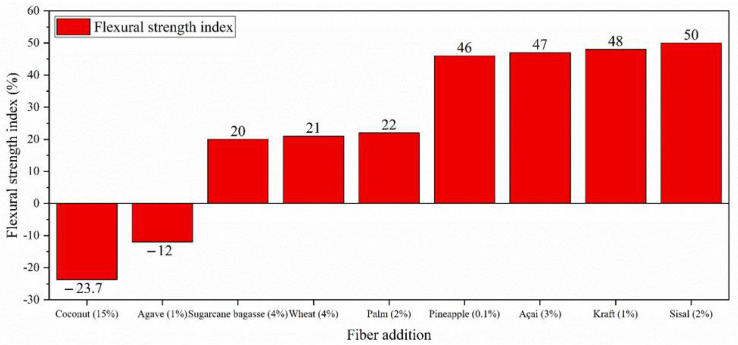
Comparative results of flexural strength of different natural fiber additions in cement composites [3,12,24,28,33,53,81].

Figure 8 presents a comparative result of the flexural strength index of cementitious composites with and without natural fiber additions. Concerning the flexural strength properties of the composites, the type of treatment applied to the fiber, the curing process, and the amount of fiber in the composition influence the composite positively or negatively. The inclusion of natural fibers usually improves the brittle structure of cement composites, providing better ductility and better post-cracking energy absorption. The energy absorption allows the fibers to transmit and redistribute the bending stresses to the matrix, depending on the adhesion tension between the fiber and the matrix.

## 6. Conclusions and Future Perspectives

After reviewing the literature, some points were observed. First, most lignocellulosic fibers have better mechanical properties after undergoing some type of treatment. The less water the fiber absorbs, the more water remains in the mortar substrate for cement hydration. The wear mechanisms of the material are also reduced since fibers that absorb a lot of water tend to deteriorate faster.

The second point concerns the proportion of fiber used in the composite mixture. In some studies, the more fiber, the better the characteristics of the composite. However, a high number of fibers absorbs a large amount of water when untreated and increases the degradation mechanisms of the material. Generally, a maximum point is reached for the inclusion of lignocellulosic fibers in the cementitious matrix. This means that up to a certain proportion, researchers achieve gains in the physical–mechanical characteristics of the composites, but if the fiber is increased significantly, the opposite effect is observed. When the amount of fibers is increased too much, the composite cannot accommodate all these fibers within it, and the wettability of the fibers for the composite is too high.

In the literature, works with lignocellulosic fibers in cementitious materials generally reach this wettability point in a range between 3% and 5% of the proportion of fibers compared to the proportion of cement used. This range differs greatly for long and short fibers. Generally, fibers that have reduced dimensions can be inserted in larger proportions. As long as these fibers do not have a dimension below the critical length, the fibers can be inserted into the matrix in high proportions providing smooth load transfer. Cases of long fibers such as kenaf, when inserted in proportions greater than 5%, run the risk of making the composite too rigid.

The third and last point is the interface between the lignocellulosic material and the matrix, which remains the main point for the success of the composite. The fiber length, surface roughness and adhesion, reduction of its hydrophilic character, fiber orientation within the matrix, and amount of cellulose directly influenced the formation of whether the composite had a good interface or not. When the interface is good, the fibers function as a more ductile element to help the concrete withstand the stresses. However, if poorly formed, they increase the number of voids in the part, causing cracks and ruptures.

This improvement in the interface is directly linked to the types of treatments that have been implemented. Mercerization is currently the most common treatment and results in good characteristics of the composite. However, the evolution in ways of treating natural fibers is the key point for future research. Treatment forms that aim to incorporate the fiber into the concrete as a fine mortar binder, as seen in the fiber cement-slag coating, seem to be good alternatives, since they managed to compact the fibers in the matrix very well, creating a good interface.

Future work on natural fibers in cementitious composites may explore other treatments compared to mercerization treatments. It was observed in the literature that few studies carry out a large number of tests for the same type of fiber. In this way, it would be possible to confirm the best types of treatment, whether alkaline treatment or not, for each type of fiber.

As this work focused on the main Brazilian fibers, it was not possible to carry out in-depth research on just one type of fiber. Future works may explore a single Brazilian fiber and carry out a complete literature review on it. In addition, further research could explore the fibers of sugarcane bagasse, pineapple, and açai.

Thus, it is concluded that the application of lignocellulosic fibers for use in cementitious materials has potential for use in coating structures due to low density and high compressive strength. The literature discussed in this work contributes to a review of the potential of Brazilian fibers for future research.

## Figures and Tables

**Table 1:** Chemical content of natural fiber.

Fiber	Cellulose (%)	Hemicellulose (%)	Lignin (%)
Cotton [30]	82.7	5.7	-
Pineapple [24]	81.0	-	12.7
Tucum Palm [31]	78.9	1.4	17.4
Hemp [30]	74.4	17.9	3.7
Bamboo [30]	73.8	12.5	10.2
Curaua [24]	73.6	9.9	7.5
Kenaf [24]	72.0	20.3	9.0
Coconut [32]	68.9	16.8	32.1
Ramie [30]	68.6	13.1	0.6
Sisal [30]	65.8	12.0	9.9
Jute [30]	64.4	12.0	11.8
Flax [30]	64.1	16.7	2.0
Sugarcane bagasse [24]	55.2	16.8	25.3
Piassava [31]	53.2	1.71	45.7
Açai [33]	46.4	17.2	31.1

**Table 2:** Cellulose content and mechanical properties of fibers.

Fiber	Tensile Strength (MPa)	Young’s Modulus (GPa)	Elongation at Break (%)	Density (g/cm^3^)
Kenaf [24]	930.0	53.0	1.6	-
Hemp [24]	690.0	70.0	1.6	1.5
Coconut [30]	593.0	6.0	30.0	1.2
Ramie [24]	560.0	24.5	2.5	1.5
Sisal [24]	511.0–635.0	9.4–22.0	2.0–2.5	1.5
Curaua [24]	500.0–1150.0	11.8	3.7–4.3	1.4
Pineapple [24]	400.0–627.0	1.4	14.5	0.8–1.6
Jute [24]	393.0–773.0	26.5	1.5–1.8	1.3
Flax [24]	345.0–1035.0	27.6	2.7–3.2	1.5
Sugarcane bagasse [24]	290.0	17.0	-	1.3
Cotton [56]	287.0–597.0	5.5–12.6	3.0–10.0	1.5–1.6
Bamboo [24]	140.0–230.0	11.0–17.0	-	0.6–1.1
Piassava [57]	134.6–142.9	1.1–4.6	6.4–21.9	1.1
Açai [33]	17.8–20.4	15.7–18.6	-	1.4

**Table 3:** The mechanical characteristics of lignocellulosic fibers common in Brazil in relation to synthetic fibers.

Fiber	Density (g/cm^3^)	Tensile Strength (GPa)	Specific Tensile Strength (σ/ρ)	Young’s Modulus (GPa)	Specific Elastic Modulus (E/ρ)
Steel Fiber [59]	7,500,000	2.5	0.0000003	190.0–210.0	0.000025
E-Glass [60]	2.6	1.8–2.7	0.69–1.04	73.0	28.1
Sugarcane bagasse [24]	1.3	0.3	0.2	17.0	13.6
Coconut [30]	1.2	0.6	0.5	6.0	5.0
Sisal [24]	1.5	0.5–0.6	0.3–0.4	9.4–22.0	6.2–14.7
Curaua [24]	1.4	0.5–1.1	0.4–7.9	11.8	8.4
Pineapple [24]	400.0–627.0	0.6	0.38–0.75	1.4	0.9–1.8
Jute [24]	1.3	0.4–0.8	0.3–0.59	26.5	20.4
Piassava [57]	1.1	0.1	0.09	1.1–4.6	1.0–4.3
Açai [33]	1.4	0.02	0.014	15.7–18.6	11.2–13.3

**Table 4:** Different natural fiber systems studied in a cement matrix.

Fiber	Fiber Addition	Fiber Treatment	Matrix	Cure
Xerophyte (Diss and Doum) [1]	0%, 0.5%, 1%, 1.5%, 2%, 3%, 4%	1% and 3% NaOH for 30 min	Portland mortar	Submerged water
Flax [5]	2%	Boiling water and coating with hydraulic binder	Portland mortar	20 ± 2 °C and 50% RH
Pineapple [12]	0%, 0.25%, 0.5%	5% NaOH for 6 h	Alkali activated mortar	-
Sisal [20]	3%	Natural	Portland mortar	Submerged water
Sisal [53]	0%, 1%, 1.5%, 2%	1% Na_2_CO_3_ for 7d	Portland concrete	-
Kraft [28]	5%	Silica fume and and NaOH	Portland concrete	Curing bath
Piassava, tucum palm, razor grass, and jute [31]	0%, 1.5%, 3.0%, 4.5%	Natural, 8% NaOH, hot water, hornfication and hybridization	Portland mortar	Submerged water and autoclave with CO_2_
Açai [33]	0%, 1.5%, 3%, 5%	Natural and 5% NaOH solution	Portland mortar	Air, 60% RH
Curauá [57]	2%	Hot water	Portland mortar	Submerged water

**Table 5:** Comparative results of the relative compressive index (RCI) for 28 days of curing for different lignocellulosic fibers with different fiber percentages.

Natural Fibers from Brazil											
Sug. Bagasse—No Treatment [77]		Coconut— No Treatment [81]		Sisal— No Treatment [65]		Açai— 5% NaOH [33]		Pineapple— 5% NaOH [125]		Piassava— CO_2_ Water [31]	
wt.% Fibers	RCI (%)	wt.% Fibers	RCI (%)	wt.% Fibers	RCI (%)	wt.% Fibers	RCI (%)	wt.% Fibers	RCI (%)	wt.% Fibers	RCI (%)
0	100.0	0	100.0	0	100.0	0	100.0	0	100	0	100.0
5	104.7	0.2	125.0	0.6	100.8	1.5	109.1	0.05	111.8	1.5	109.2
10	111.6	0.4	100.0	1.2	103.3	3	120.2	0.1	118.2	3	98.1
15	120.9	0.6	100.0	1.8	99.2	4.5	111.9	0.15	111.8	4.5	109.4
20	97.7	0.8	115.0					0.2	108.8		

## Data Availability

Not applicable.
